# Carotid sinus nerve electrical stimulation in *conscious* rats attenuates systemic inflammation via chemoreceptor activation

**DOI:** 10.1038/s41598-017-06703-0

**Published:** 2017-07-24

**Authors:** Fernanda Machado Santos-Almeida, Gean Domingos-Souza, César A. Meschiari, Laura Campos Fávaro, Christiane Becari, Jaci A. Castania, Alexandre Lopes, Thiago M. Cunha, Davi J. A. Moraes, Fernando Q. Cunha, Luis Ulloa, Alexandre Kanashiro, Geisa C. S. V. Tezini, Helio C. Salgado

**Affiliations:** 1 Department of Physiology, Ribeirão Preto Medical School – University of São Paulo, Ribeirão, Preto 14049-900 Brazil; 2 Department of Pharmacology, Medical School of Ribeirão Preto – University of São Paulo, Ribeirão, Preto 14049-900 Brazil; 3 Center of Immunology and Inflammation. Rutgers– New Jersey Medical School, Rutgers University, Newark, NJ 07103 USA

## Abstract

Recent studies demonstrated a critical functional connection between the autonomic (sympathetic and parasympathetic) nervous and the immune systems. The carotid sinus nerve (CSN) conveys electrical signals from the chemoreceptors of the carotid bifurcation to the central nervous system where the stimuli are processed to activate sympathetic and parasympathetic efferent signals. Here, we reported that chemoreflex activation via electrical CSN stimulation, in conscious rats, controls the innate immune response to lipopolysaccharide attenuating the plasma levels of inflammatory cytokines such as tumor necrosis factor (TNF), interleukin 1β (IL-1β) and interleukin 6 (IL-6). By contrast, the chemoreflex stimulation increases the plasma levels of anti-inflammatory cytokine interleukin 10 (IL-10). This chemoreflex anti-inflammatory network was abrogated by carotid chemoreceptor denervation and by pharmacological blockade of either sympathetic - *propranolol* - or parasympathetic - *methylatropine* – signals. The chemoreflex stimulation as well as the surgical and pharmacological procedures were confirmed by real-time recording of hemodynamic parameters [pulsatile arterial pressure (PAP) and heart rate (HR)]. These results reveal, in conscious animals, a novel mechanism of neuromodulation mediated by the carotid chemoreceptors and involving both the sympathetic and parasympathetic systems.

## Introduction

Classically, the immune system is considered to be modulated by humoral factors such as cytokines, catecholamines, and hormones. However, recent studies have shown specific neuronal networks connecting the nervous and the immune system^1, 2^. For example, afferent (sensory) and efferent (effector) vagus nerve fibers connect the brain with the peripheral immune system^3–5^. Given that the vagus nerve is a cholinergic network producing acetylcholine, this connection was named the *“cholinergic anti-inflammatory pathway”* for its potential to inhibit systemic inflammation in endotoxemia^3–6^. In addition to the parasympathetic vagus nerve^3, 6, 7^, a recent study proposed a sympathetic regulation of the immune system^8^. These authors proposed a *“splanchnic anti-inflammatory pathway”* modulating systemic inflammation in experimental endotoxemia. The intravenous administration of Escherichia coli lipopolysaccharide (LPS) to anesthetized rats, triggered a strong systemic inflammatory response under the control of the splanchnic nerves, which arise from the sympathetic trunk in the thorax and innervates the viscera^8^.

One critical limitation of these previous studies of neuro-immune modulation is that they were performed in *anesthetized* animals^3, 6^. However, anesthetics causes undesirable collateral effects on both the nervous^9^ and the immune systems^10^. For instance, anesthetized animals are influenced, to an unknown extent, by the direct effects of general anesthesia and the elimination of higher central nervous system control of the cardiovascular system^9^. Accordingly, these collateral effects significantly attenuate the arterial baroreflex^11–15^. This detrimental effect of anesthesia motivated our laboratory to investigate the reflex control of the cardiovascular system in conscious animals^16–22^. Thus, the experience of our laboratory on neural cardiocirculatory control in conscious animals (rats and mice)^3, 6, 8^ allow us to study neuronal regulation of inflammation in conscious animals without the interference of anesthesia.

We recently developed a novel technique to electrically activate the carotid sinus nerve (CSN) in conscious rats^11^. This technique allows us to perform a simultaneous activation of both the carotid baro- and chemoreflex. The carotid baroreflex activates the parasympathetic and inhibits the sympathetic drive, whereas the carotid chemoreflex activates both the sympathetic and parasympathetic drive^20, 23^. Electric CSN-stimulation combined with selective denervation of the carotid chemoreceptors^20, 24, 25^ allows us to define the relative role of the sympathetic and parasympathetic system in modulating systemic inflammation.

Based on previous studies, we hypothesized that electrical activation of the chemoreflex will attenuate LPS-induced systemic inflammation throughout the sympathetic and parasympathetic systems. To test this hypothesis, conscious rats underwent electrical CSN-stimulation combined with selective bilateral surgical denervation of the carotid chemoreceptors^20, 24, 25^. In addition to these surgical procedures, we also used pharmacological approaches including methylatropine and propranolol to evaluate the parasympathetic and the sympathetic systems, respectively.

## Results

**Figure 1:**
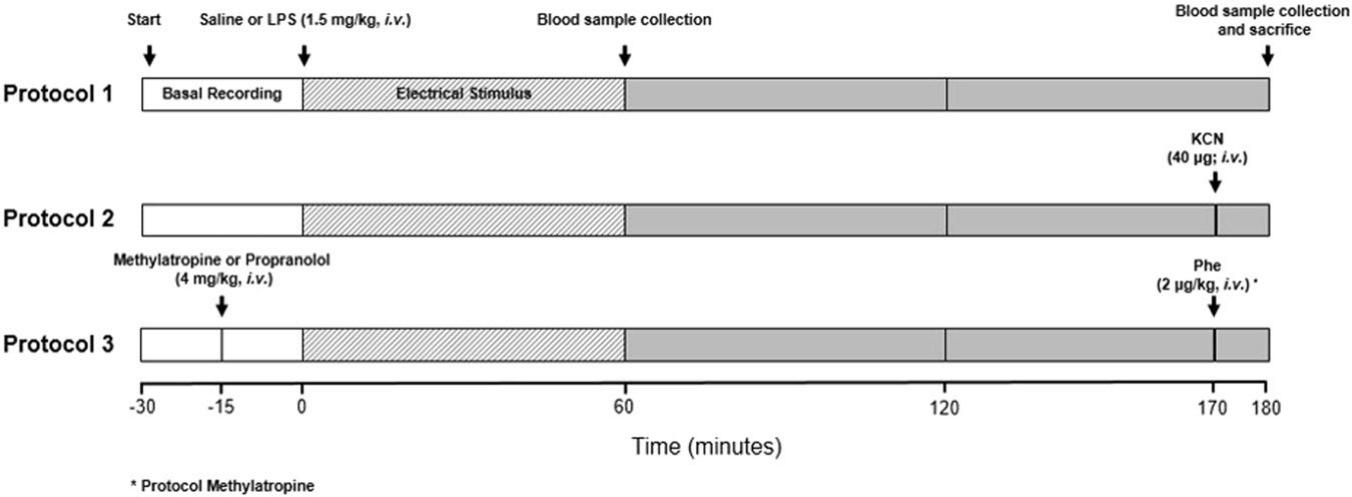
Experimental Protocols. White bars represent the recording period before the stimulation; hatched bars represent the timeframe under electrical stimulation. After the electrical stimulation, the animals were monitored for another 2 hours (gray bars). Saline (vehicle) or LPS (1.5 mg/kg, *i.v*.) were injected at time 0 in all protocols.

Figure 1 illustrates the experimental protocols and the procedures conducted. Animals were monitored up to 3 hours after the electrical stimulation.

### Carotid sinus nerve stimulation attenuated LPS-induced systemic inflammation

**Figure 2:**
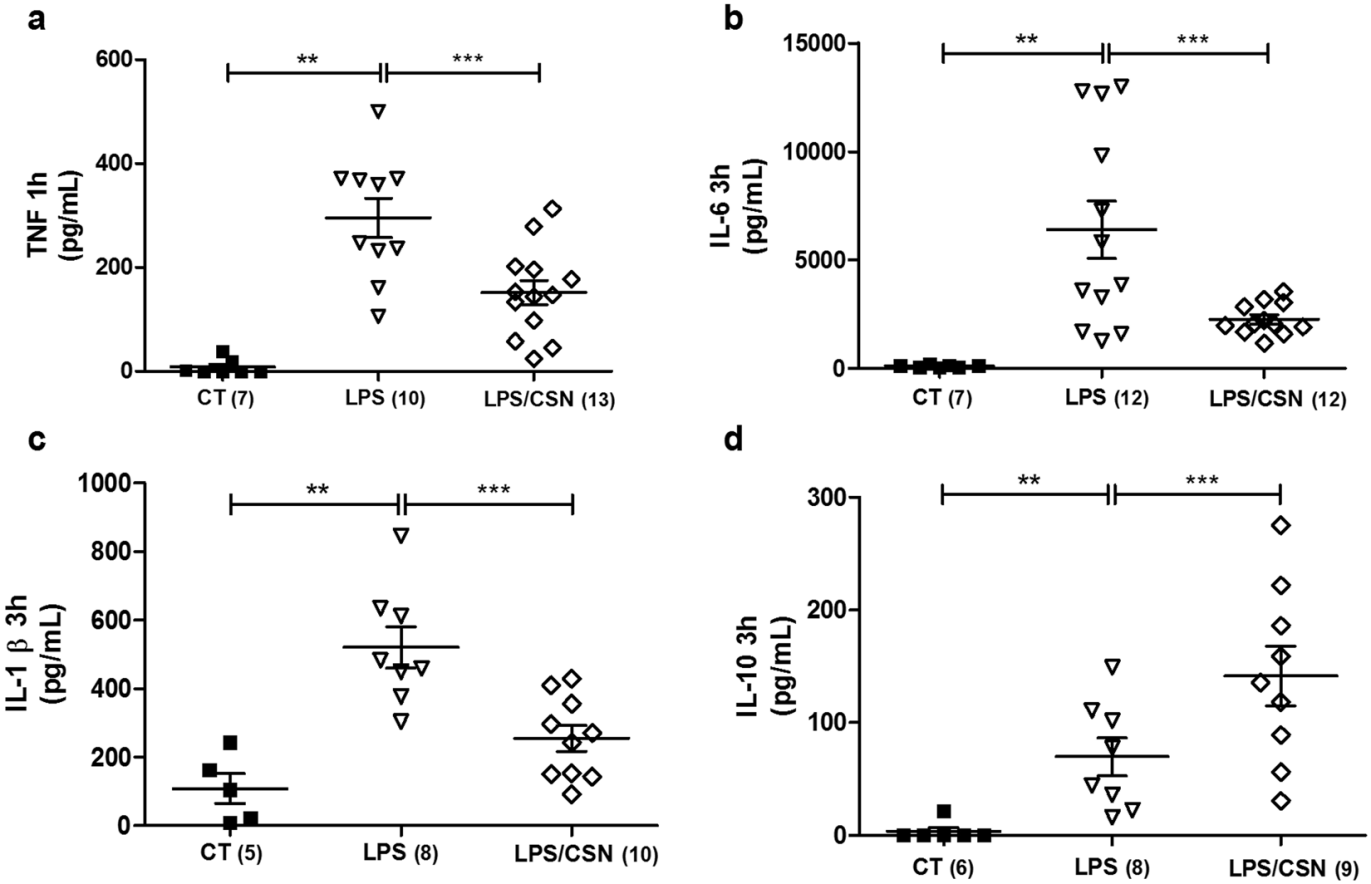
Effect of electrical stimulation of the carotid sinus nerve (CSN) on plasma cytokines concentration in LPS induced systemic inflammation in conscious rats. Panel *a*: TNF (**p < 0.001 vs Control, ***p = *0.002 vs LPS)* 1 hour after LPS administration. Panels *b*, *c*, and *d, respectively*: IL-6 (**p < 0.001 vs Control, ***p = *0.007 vs LPS*), IL-1β (**p < 0.001 vs Control, ***p = *0.005 vs LPS*), and IL-10 (**p < 0.001 vs Control, ***p = *0.0019 vs LPS*) 3 hours after LPS administration. Number of animals is shown between parentheses.

First, we investigated whether electrical CSN activation in *conscious* rats, which activates both the baro- and chemoreflex, regulates the LPS-induced systemic inflammation. Systemic inflammation was determined by the concentration of inflammatory cytokines in the plasma. Previous studies analyzed the anti-inflammatory potential of the vagus nerve in anesthetized animals, and little is known about the neuromodulation of the innate immunity in *conscious* animals. Our results show that CSN stimulation significantly attenuated the LPS-induced plasma levels of inflammatory cytokines including TNF, IL-1β, and IL-6. This is a selective effect because CSN activation did not inhibit but it significantly increased the plasma levels of anti-inflammatory cytokine IL-10 as compared with the sham treatment (Fig. 2a–d). Plasma levels of IL-1β, IL-6 and IL-10 did not change significantly (data not shown) at the first hour after LPS administration.

### Chemoreceptor denervation prevented the anti-inflammatory effect of CSN stimulation

**Figure 3:**
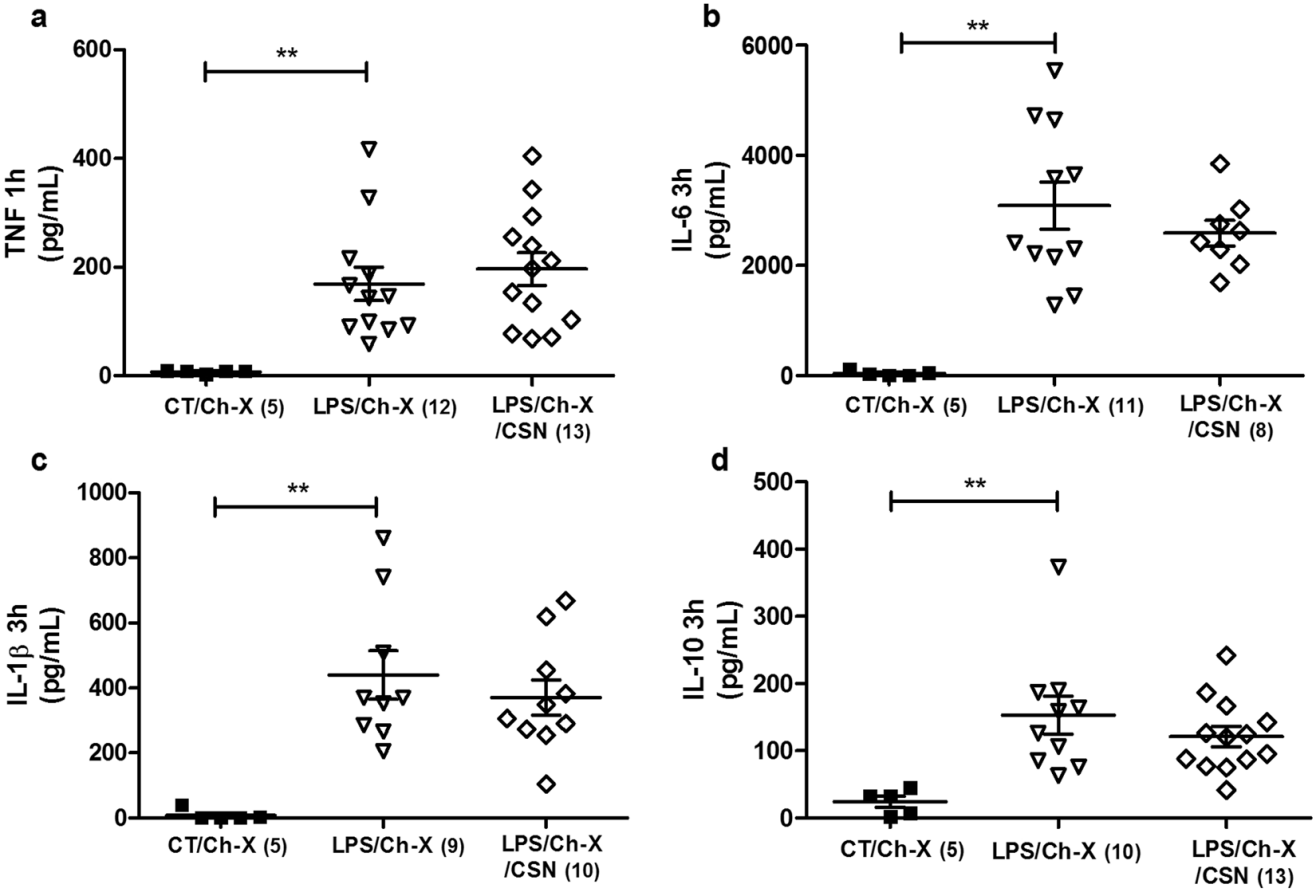
Carotid body denervation (Ch-X) abrogates the anti-inflammatory effects of electrical stimulation of the carotid sinus nerve (CSN) on plasma cytokines concentration in LPS induced systemic inflammation in conscious rats. Panel *a*: TNF (**p = *0.005 vs Control*) 1 hour after LPS administration. Panels *b*, *c*, and *d, respectively*: IL-6 (**p < *0.001 vs Control*), IL-1β (**p < *0.001 vs Control*), and IL-10 (**p = *0.004 vs Control*) at 3 hours after LPS administration. Number of animals is shown between parentheses.

We next performed bilateral surgical carotid body denervation to determine whether the anti-inflammatory effect of CSN stimulation depends on the chemoreflex. Carotid sinus denervation abolished the anti-inflammatory effect of CSN stimulation to inhibit LPS-induced plasma inflammatory cytokines (Fig. 3a–c) and prevented the potential of CSN stimulation to induce IL-10 (Fig. 3d). These findings highlight the role of the chemoreflex in this neuromodulatory pathway and its potential to control systemic inflammation.

### Parasympathetic blockade abolished the anti-inflammatory effect of CSN stimulation

**Figure 4:**
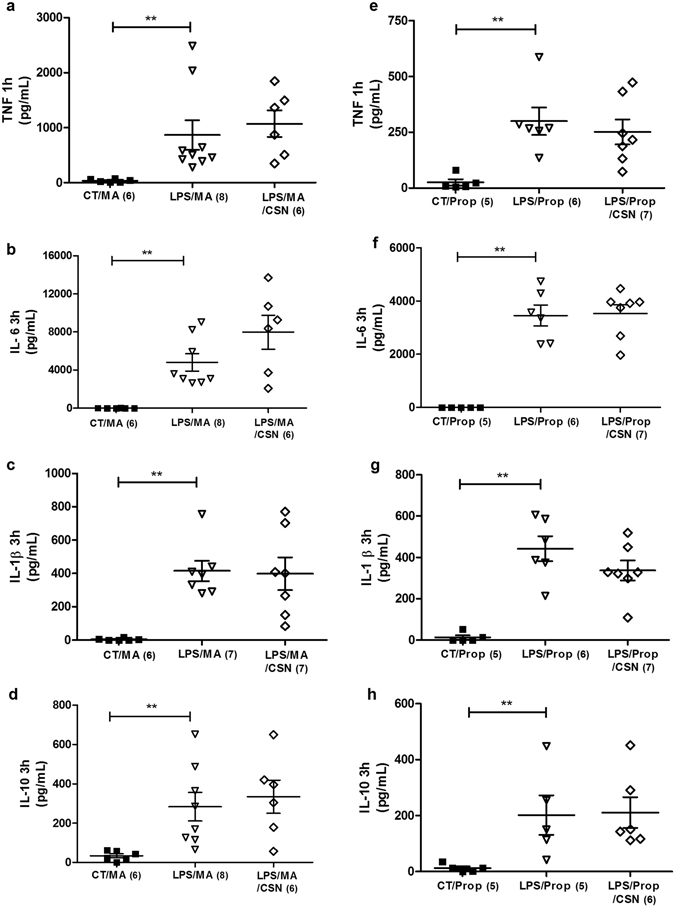
Methylatropine (MA) and Propranolol (Prop) administration abrogates the anti-inflammatory effect of electrical stimulation of the carotid sinus nerve (CSN) on plasma cytokines concentration in LPS induced systemic inflammation in conscious rats. Panel *a*: TNF (**p = *0.02 vs Control*) 1 hour after LPS and MA administration. Panels *b*, *c*, and *d*, respectively: IL-6 (**p = *0.007 vs Control*), IL-1β (**p = *0.002 vs Control*) and IL-10 (**p = *0.01 vs Control*) at 3 hours after LPS and MA administration. Panel *e*: TNF(**p = *0.009 vs Control*) 1 hour after LPS and Prop administration. Panels *f*, *g*, and *h, respectively*: IL-6 (**p < *0.001 vs Control*), IL-1β (**p < *0.001 vs Control*), and IL-10 (**p = *0.04 vs Control*) at 3 hours after LPS and Prop administration. Number of animals is shown between parentheses.

Because the chemoreflex simultaneously activates both sympathetic and parasympathetic activity^20, 23^, we performed the chemoreflex activation in rats pre-treated with methylatropine to block peripheral muscarinic receptors and inhibit the parasympathetic system^26^. Our results showed that methylatropine, a classical muscarinic antagonist, abolished the inhibitory effect of CSN stimulation on inflammatory cytokines (TNF, IL-1β, and IL-6) and the induction of IL-10 (Fig. 4a–d). Figure 4a shows that methylatropine potentiates, by itself, the LPS-induced TNF plasma levels (Fig. 2a). These findings demonstrate that the modulatory effect of the chemoreflex in LPS-induced systemic inflammation involves muscarinic parasympathetic activation.

### Sympathetic blockade abolished the anti-inflammatory effect of CSN stimulation

To investigate the role of the sympathetic drive in the chemoreceptor anti-inflammatory network, we performed electrical CSN stimulation in rats pre-treated with propranolol to block sympathetic activity^26^. Our results show that β-adrenergic receptors blockade abolished all the effects of CSN stimulation preventing the inhibition of inflammatory cytokines as well as the induction of IL-10 (Fig. 4e–h). These findings demonstrate that the modulatory effect of the chemoreflex in LPS-induced systemic inflammation is also mediated by sympathetic activation.

## Discussion

This study shows that electrical CSN stimulation attenuated the innate immune response to bacterial LPS in *conscious* rats decreasing the plasma levels of the inflammatory cytokines (TNF, IL-1β, and IL-6). Moreover, CSN stimulation induced a different effect in anti-inflammatory cytokines enhancing the production of IL-10. We also show that the anti-inflammatory effect of CSN stimulation depends on the integrity of the chemoreceptors associated with sympathetic and parasympathetic activation.

The present study is based on CSN stimulation performed in unanesthetized, conscious freely moving animals. Usually, the anti-inflammatory potential of nerve stimulation (e.g. vagus nerve) were investigated in anesthetized animals^3, 6^. However, anesthesia affects critical physiological mechanisms including the baroreflex^27^, the immune response and the nervous system^28^. Indeed, a previous study showed that anesthetics have significant anti-inflammatory effects by decreasing inflammatory cytokines^28^. Altogether, these studies suggest that therapeutic strategies in *anesthetized* animals may not reproduce the normal physiological scenario of *conscious* animals. Therefore, the absence of anesthesia in our protocol mimics physiological homeostasis, providing significant advantages for the study of novel therapeutic strategies using electrical nerve stimulation.

Previous studies proposed two potential mechanisms of neuromodulation: one primarily mediated by the parasympathetic system^3, 4^ and the second mediated by the sympathetic drive^8^. This present study provides support for the involvement of these two branches of the autonomic nervous system. The efferent vagal cholinergic signaling represents a connection from the brain to the peripheral immune system, comprising the so called “**cholinergic anti-inflammatory pathway**”^3, 6^ and the “**splanchnic anti-inflammatory pathway** mediated by the parasympathetic and sympathetic nervous systems, respectively^8^. The following anatomo-physiological considerations provide support to our conclusion. First, the carotid sinus is a special vascular anatomical structure containing barosensitive nerve endings - baroreceptors - that respond to deformation or the strain of the vessel walls^29^, and chemical sensors – chemoreceptors - able to detect hypoxemia, hypercapnia and hydrogen ions in the blood^23^. When activated, the baroreceptors inhibit sympathetic activity and increase parasympathetic signals to the heart and other tissues^30, 31^. Second, the chemoreceptors activate both the sympathetic and parasympathetic branches of the autonomic nervous system^23, 32^. When we examined the effect of both the baro- and chemoreceptors on hemodynamic parameters (pulsatile arterial pressure (PAP), and heart rate (HR)) after CSN stimulation, we found that the carotid chemoreceptor activation attenuated the hypotensive and totally blocked the bradycardic response. These findings demonstrated a predominance of sympathetic activation elicited by the chemoreflex, overcoming the sympathetic inhibition triggered by the carotid baroreflex^20^.

In addition to their role regulating arterial blood homeostasis, the carotid chemoreceptors can also act as peripheral sensors of immunogenic agents in the blood. The carotid chemoreceptor neural pathway is proposed as a peripheral sensor for the presence of immunogenic agents in the blood, i.e., several manifestations of endotoxemia could be mediated by incoming neural signals from the carotid chemoreceptor pathway for immune-to-brain communication^33^. Moreover, Fernandez *et al*.^34^ proposed that carotid activation increases sympathetic activity and adrenal glucocorticoids release and that electrical stimulation of peripheral chemoreceptors may be a suitable therapeutic approach for regulating inflammatory disorders.

In the present study, we electrically stimulated the CSN and attenuated LPS-induced inflammatory responses by activating the peripheral - carotid – chemoreflex. Ultimately, this procedure activated both autonomic systems: the parasympathetic – vagal – activity as confirmed by intravenous methylatropine administration, as well as the sympathetic activity confirmed with propranolol administration. The administration of methylatropine potentiated the effect of LPS on TNF plasma levels, indicating that even a basal parasympathetic activity is capable of modulating the inflammatory response.

Mechanistically, we propose that noradrenaline and acetylcholine released after the CSN stimulation can inhibit the production of inflammatory cytokines in macrophages, the main cellular source of TNF in endotoxemia^3, 35^. However, an integrated link coordinating both autonomic systems to control inflammation could explain our experimental data^36^. In this study, Vida *et al*.^36^ demonstrated that acetylcholine released from the vagus (parasympathetic) nerve into the celiac ganglia stimulates the splenic (sympathetic) nerve, inducing the splenic production of norepinephrine. In addition to the inhibition of inflammatory cytokines, we showed that chemoreflex activation increased plasma IL-10 levels. Our results concur with previous studies showing the capacity of adrenergic agonists to induce IL-10, a potent anti-inflammatory cytokine able to inhibit TNF production^37, 38^.

In summary, we demonstrated that electrical CSN stimulation prevented LPS-induced systemic inflammation in *conscious* rats via a neural mechanism dependent on the integrity of peripheral - carotid - chemoreceptors, involving both sympathetic and parasympathetic activation. Further, taking into account that carotid body denervation (Ch-X) abrogates the anti-inflammatory effects of electrical stimulation of the carotid sinus nerve (CSN) in conscious rats (Fig. 3), this finding precludes an antiinflammatory role played by the carotid baroreflex in the inflammatory response to LPS.

The development of methods to stimulate CSN (and more specifically the neural pathways involving chemoreceptors activation) may be a promising therapeutic strategy to suppress cytokine-mediated inflammatory diseases such as sepsis and auto-immune diseases.

## Methods

### Animals

All animal care and experimental procedures were in accordance with the Ethical Principles in Animal Research adopted by the National Council for the Control of Animal Experimentation (CONCEA) and were approved by the Local Animal Ethical Committee from Ribeirão Preto Medical School of the University of São Paulo, protocol #171/2013. All methods were carried out in accordance with The Principles of Laboratory Animal Care (NIH publication no. 85Y23, revised 1996)”.

### Animal experiments

Male Wistar rats (250–280 g) obtained from the Main Animal Facility of Ribeirão Preto Medical School (University of São Paulo) were housed in plastic cages under a 12-h light/dark cycle (lights on at 7 am) at 20 °C ± 1 °C and maintained in groups of five per cage (40 × 33 × 18 cm). The animals had unrestricted access to food and tap water. The number of animals used was the minimum required to ensure the reliability of the results, and every effort was made to minimize animal discomfort.

### Surgical procedures

Animals were anesthetized with a mixture of ketamine (50 mg/kg, *i.p*., União Química Farmacêutica Nacional S/A, Embu-Guaçu, SP, Brazil) and xylazine (10 mg/kg, *i.p*., Hertape Calier Saúde Animal S/A, Juatuba, MG, Brazil) and placed on a surgical table. After anterior neck incision under a surgical microscope view (M902MFZ, DFV com. Ind. Ltda, São Paulo, Brazil) the left carotid sinus was carefully isolated. A pair of flexible stainless steel electrode was placed around the internal carotid artery, separated by approximately 2 mm, and isolated from adjacent structures with a cold-polymerizing resin (Kwik-Sil, World Precision Instruments Inc., Sarasota, Florida, USA) according to the technique described by Katayama *et al*.^20^ elsewhere. Flexible wires connected to the electrodes were exteriorized in the interscapular region of rats. A group of animals had bilateral denervation of the carotid chemoreceptors by means of the ligation of the carotid body artery by a technique adapted from Franchini and Krieger^24^. All surgical procedures were performed under aseptic conditions and all the material were implanted after proper sterilization.

During surgical implantation of the electrodes, the rats were also instrumented with polyethylene catheters (PE-50 soldered to PE-10; Intramedic, Clay Adams, Parsippany, NJ, USA) into the femoral artery and vein, for both the direct measurement of AP and for the drug administration, respectively. The catheters were tunneled subcutaneously and exteriorized in the nape of the neck, and the surgical incision sites were sutured. The animals were allowed to recover in individual cages until the next day.

At the end of surgery, all animals received an injection of a polyvalent veterinary antibiotic (Pentabiótico, 0.2 mL, *i.m*.; Fort Dodge, Campinas, SP, Brazil) and an injection of the anti-inflammatory and analgesic flunixin meglumine (Banamine, 25 mg/kg, *s.c*.; Schering-Plough, Cotia, SP, Brazil). No significant postoperative weight loss or mortality was observed in the animals.

### Experimental Protocols

#### Protocol 1

Data recordings were carried out in *conscious and unrestrained* rats one day after surgery. The basal AP was recorded during 30 min before starting CSN stimulation. After basal recordings, animals received saline (*i.v*.) or Escherichia coli Lipopolysaccharide (LPS) (1.5 mg/kg, *i.v*.) to induce systemic inflammation (LPS administration induced a significant reduction in mean AP; see Supplementary Fig. S1). Immediately after LPS or saline administration, a long-lasting (60 min)^21^, electrical stimulus (1 mA, 1ms, 30 Hz) was applied and the animals were monitored for 3 hours. These parameters of stimulation were based in previous studies^20, 21^ from our laboratory. The effects of CSN stimulation on the hemodynamic parameters showing the effectiveness of electrical activation are included in Supplement (see Supplementary Fig. S2). During all CSN-stimulation, AP and HR were recorded. Arterial blood was withdrawn in heparinized tubes and centrifuged (15 min at 3.500 rpm, 4 °C), for plasma extraction, on the first and third hour after saline or LPS administration. The volume was replaced (1 mL) by means of intravenous administration of sterile saline 0.9%. All plasma samples were immediately frozen in liquid nitrogen and stored at −80 °C for enzymatically linked immunosorbent assay (ELISA).

#### Protocol 2

**Figure 5:**
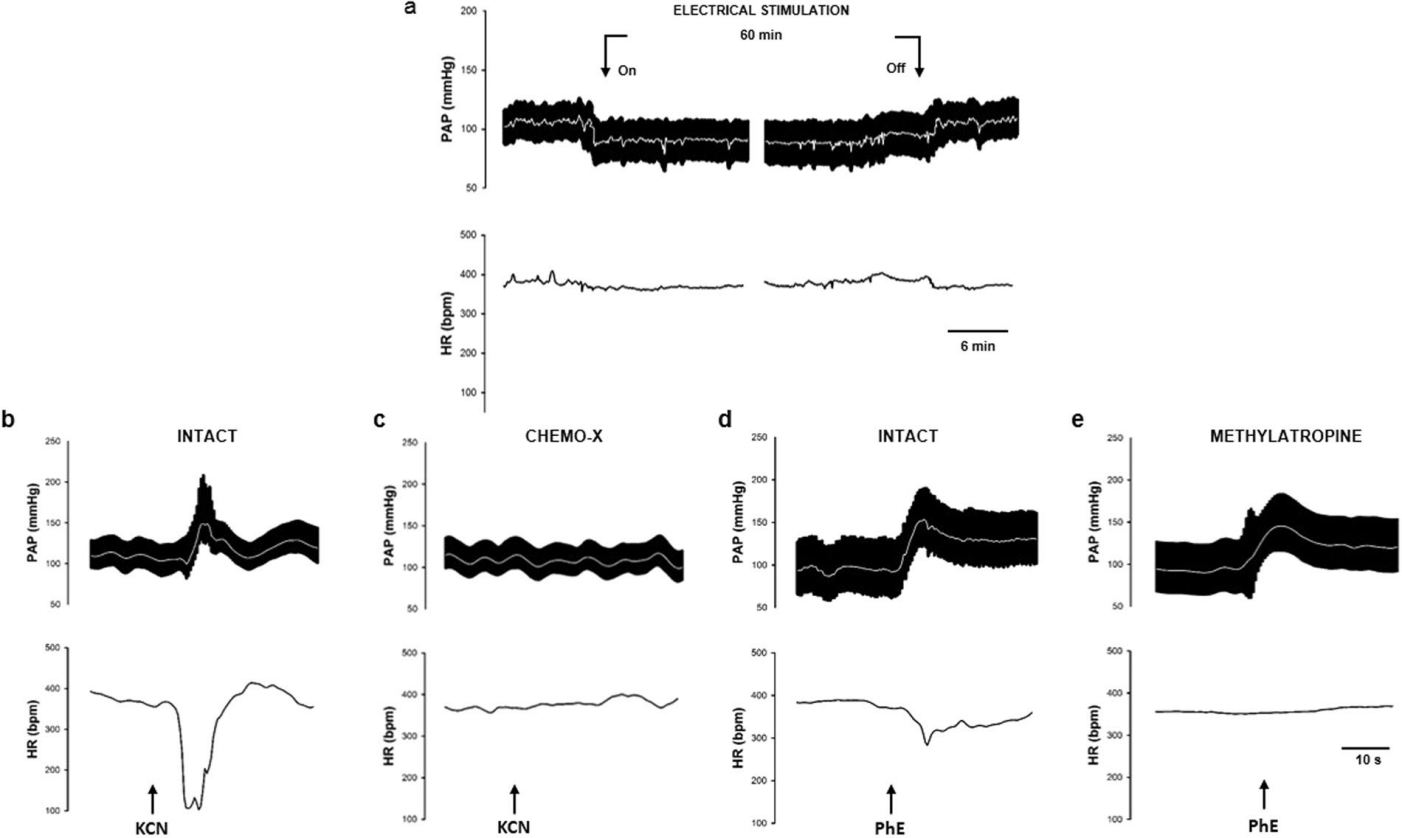
Hemodynamic responses to electrical stimulation of the carotid sinus nerve. Panel *a*: Tracings of pulsatile arterial pressure (PAP) [white line represents mean AP] and heart rate (HR) before, during and right after turning off the electrical stimulation. Panels *b* and *c*: Hemodynamic responses to KCN in intact and carotid body denervated rats. Panels *d* and *e*: effect of phenylephrine (PhE) in intact rats without and under methylatropine administration, respectively.

To evaluate the role of the chemoreceptors in modulating inflammation, bilateral carotid chemoreceptors denervation was performed as described in surgical procedures. The control group have the chemoreceptors deactivated and received neither LPS nor stimulation of CSN. The effectiveness of denervation was confirmed by the absence of the chemoreflex responses (hypertension and bradycardia) after the intravenous injection of 100 μl potassium cyanide (KCN; 40 μg per rat) 10 minutes before the end of the experimental protocol (Fig. 5).

#### Protocol 3

Since the chemoreflex simultaneously activates both sympathetic and parasympathetic peripheral activity^20, 23^, we combined the electric CSN-stimulation with either methylatropine (4 mg/kg, *i.v*.) or propranolol (4 mg/kg, *i.v.)* to specifically block parasympathetic or sympathetic activity, respectively^26^. In this protocol, the control group received only methylatropine or propranolol besides saline. The effectiveness of parasympathetic blockade was confirmed by the absence of reflex bradycardia due to the increase in the AP elicited by the administration of phenylephrine (2 mg/kg, *i.v*.) 10 minutes before the end of the experimental protocol (Fig. 5). The effectiveness of β-adrenergic receptors blockade with propranolol was confirmed by the prompt fall in AP and HR (see Supplementary Fig. S3).

### Measurement of arterial pressure

The arterial catheter was connected to a pressure transducer (MLT0380/D, ADInstruments, Sydney, Australia) and the AP signal was amplified (ML110, ADInstruments, Sydney, Australia) and sent to an IBM/PC connected to a Power Lab (ML866, ADInstruments, Sydney, Australia). The AP signal was continuously sampled (2 kHz). Mean AP and HR were calculated from the pulsatile AP.

### Cytokines assay

On the day of the assay, samples were defrosted and maintained in ice. Plasma was used to measure the levels of tumor necrosis factor (TNF), interleukin (IL)-1β, IL-6, and IL-10 by ELISA using Duo set kits from R&D Systems (Minneapolis, MN, USA) as described in Bassi *et al*.^19^. The results were expressed as pg/mL of TNF, IL-1β, IL-6, and IL-10, based on standard curves.

### Statistical analysis

The results are shown as mean ± S.E.M. (standard error of the mean). Data were compared using one-way analysis of variance (ANOVA) followed by the Newman-Keuls post hoc test, and Student’s *t*-test. Differences were considered significant when *p* < *0.05*. All statistical tests were performed with SigmaStat 3.5 software (Systat Software Inc., San Jose, CA, USA).

## Electronic supplementary material

Supplementary information
