# Supplementary material for: Carotid sinus nerve electrical stimulation in conscious rats attenuates systemic inflammation via chemoreceptor activation
Source: Sci Rep. 2017 Jul 24;7:6265. doi: 10.1038/s41598-017-06703-0 (PMC5524712; doi:10.1038/s41598-017-06703-0)
Supplement: Supplementary file 1 — Supplementary information [file 41598_2017_6703_MOESM1_ESM.pdf]

Carotid sinus nerve electrical stimulation in *conscious* rats attenuates systemic inflammation via chemoreceptor activation

Fernanda Machado Santos-Almeida[1,*]; Gean Domingos-Souza[1,*]; César A. Meschiari[1]; Laura Campos Fávaro[1]; Christiane Becari[1], Jaci A. Castania[1]; Alexandre Lopes[2]; Thiago M. Cunha[2]; Davi J. A. Moraes[1]; Fernando Q. Cunha[2]; Luis Ulloa[3]; Alexandre Kanashiro[2]; Geisa C. S. V. Tezini[1] & Helio C. Salgado[1].

[1]Department of Physiology, Ribeirão Preto Medical School – University of São Paulo, Ribeirão Preto, 14049-900, Brazil. [2]Department of Pharmacology, Medical School of Ribeirão Preto – University of São Paulo, Ribeirão Preto, 14049-900, Brazil.[3]Center of Immunology and Inflammation. Rutgers University – New Jersey Medical School, Newark, NJ 07103, USA.

Correspondence and requests for materials should be addressed to H.C.S. (email: hcsalgado@fmrp.usp.br).

*These authors contributed equally to this work.

Supplementary Figure S1

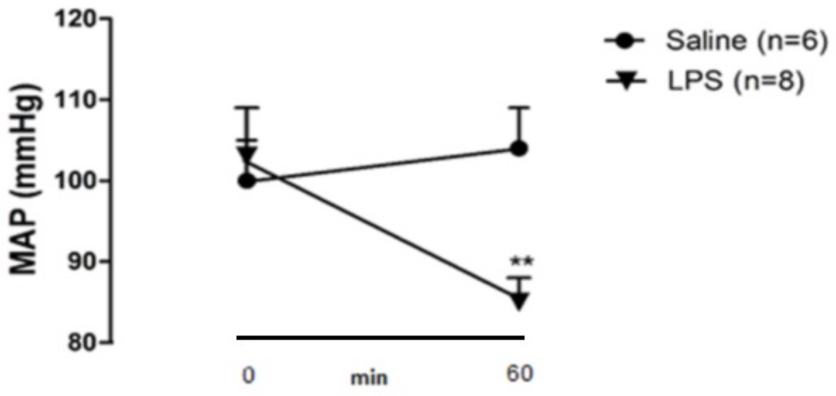

Figure S1. Mean arterial pressure (MAP) before (zero) and 60 minutes after intravenous administration of saline or Escherichia coli lipopolysaccharide (LPS). Data are expressed as the mean ± standard error of the mean; **p=0.01 compared to saline. In parentheses the number of animals.

Supplementary Figure S2

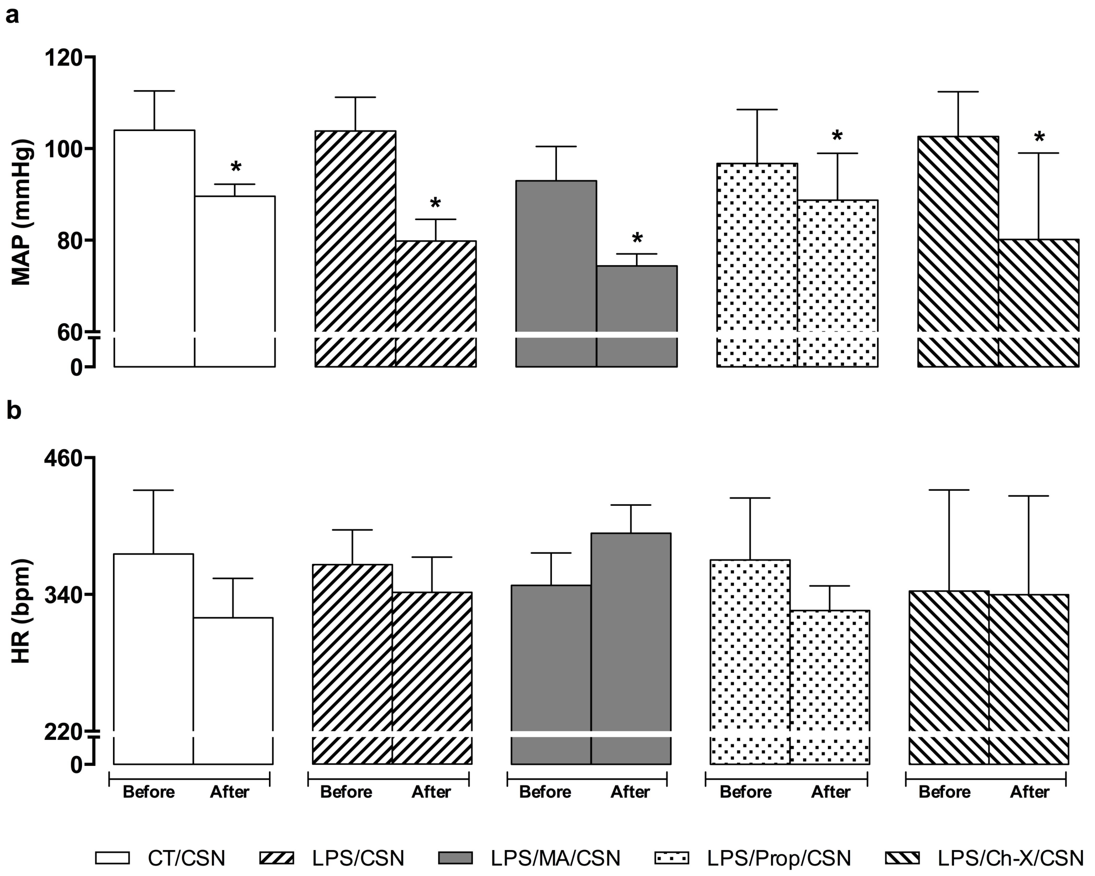

Figure S2. Hemodynamic responses [(MAP: mean arterial pressure; panel a) and HR: heart rate; panel b)] displaying the efficacy of electrical activation of the carotid sinus nerve in promoting a prompt fall in MAP in the different groups. **CT/CSN**: Control plus Carotid Sinus Nerve stimulation; n=5; **LPS/CSN**: Escherichia coli lipopolysaccharide (LPS) plus Carotid Sinus Nerve stimulation; n=7; **LPS/MA/CSN**: LPS plus Methylatropine administration and Carotid Sinus Nerve stimulation; n=5; **LPS/Prop/CSN**: LPS plus Propranolol administration and Carotid Sinus Nerve stimulation; n=5; **LPS/Ch-X/CSN**: LPS plus chemoreceptor denervation and Carotid Sinus Nerve stimulation; n=7. *Stimulation factor $p<0.0001$.

Supplementary Figure S3

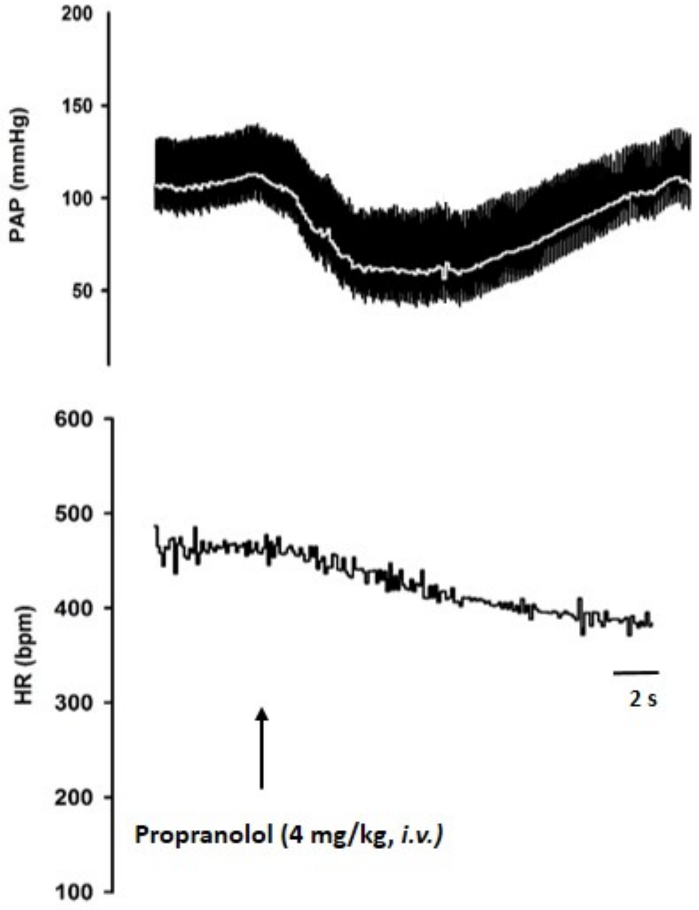

Figure S3. Tracings of pulsatile arterial pressure (PAP) [white line represents mean arterial pressure] and heart rate (HR) before and right after intravenous administration of propranolol in a conscious rat.
